# Caveolin-1 sensitizes rat pituitary adenoma GH3 cells to bromocriptine induced apoptosis

**DOI:** 10.1186/1475-2867-7-1

**Published:** 2007-03-02

**Authors:** Yan-Nian Jiang, Yi-Hung Li, Meng-Wei Ke, Ting-Yu Tseng, Yueh-Bih Tang, Mu-Chiou Huang, Winston Teng-Kuei Cheng, Yu-Ten Ju

**Affiliations:** 1 Department of Animal Science and Technology, National Taiwan University, Taipei, Taiwan; 2 Division of Plastic Surgery, Department of Surgery, National Taiwan University Hospital, Taipei, Taiwan; 3 Department of Animal Science, National Chung Hsing University, Taichung, Taiwan

## Abstract

**Background:**

Prolactinoma is the most frequent pituitary tumor in humans. The dopamine D_2 _receptor agonist bromocriptine has been widely used clinically to treat human breast tumor and prolactinoma through inhibition of hyperprolactinemia and induction of tumor cell apoptosis, respectively, but the molecular mechanism of bromocriptine induction of pituitary tumor apoptosis remains unclear. Caveolin-1 is a membrane-anchored protein enriched on caveolae, inverted flask-shaped invaginations on plasma membranes where signal transduction molecules are concentrated. Currently, caveolin-1 is thought to be a negative regulator of cellular proliferation and an enhancer of apoptosis by blocking signal transduction between cell surface membrane receptors and intracellular signaling protein cascades. Rat pituitary adenoma GH3 cells, which express endogenous caveolin-1, exhibit increased apoptosis and shrinkage after exposure to bromocriptine. Hence, the GH3 cell line is an ideal model for studying the molecular action of bromocriptine on prolactinoma.

**Results:**

The expression of endogenous caveolin-1 in GH3 cells was elevated after bromocriptine treatment. Transiently expressed mouse recombinant caveolin-1 induced apoptosis in GH3 cells by enhancing the activity of caspase 8. Significantly, caveolin-1 induction of GH3 cell apoptosis was sensitized by the administration of bromocriptine. Phosphorylation of caveolin-1 at tyrosine 14 was enhanced after bromocriptine treatment, suggesting that bromocriptine-induced phosphorylation of caveolin-1 may contribute to sensitization of apoptosis in GH3 cells exposed to bromocriptine.

**Conclusion:**

Our results reveal that caveolin-1 increases sensitivity for apoptosis induction in pituitary adenoma GH3 cells and may contribute to tumor shrinkage after clinical bromocriptine treatment.

## Background

Prolactinomas, prolactin-secreting pituitary adenomas, are the most frequently found functional pituitary tumors in humans. The pathology of prolactinomas involves dysfunction of lactotrophic cells in the anterior pituitary gland which in turn leads to hyperprolactinemia [1]. First line therapy for prolactinomas includes management with dopamine agonists. Bromocriptine is commonly chosen as a therapeutic agent for patients with prolactinomas or other pituitary adenomas; bromocriptine binds to the dopamine D_2 _receptor on pituitary epithelial cells to inhibit prolactin secretion [2]. Treatment with bromocriptine causes tumor shrinkage [3]. A rat pituitary prolactinoma cell line, GH3 is commonly used as a cellular model for studying prolactinoma formation. Long-term incubation of GH3 with bromocriptine induces cell apoptosis [4].

Caveolin-1 is a 21–24 kDa major integral membrane protein on caveolae, an invaginated structure on cellular membranes enriched with high numbers of cholesterol, glycosphingolipid and signaling molecules [5]. Caveolin-1 has been suggested to negatively regulate many different signaling molecules located on caveolae via mutual interactions that compartmentalize the signaling molecules and suppress cell growth [6]. Caveolin-1 is functionally involved in endocytosis, transcytosis, cholesterol transport, homeostasis, negative regulation of Ras-, NO-, and G-protein-coupled-receptors, and growth-factor-mediated protein kinase signaling cascades [7-9].

There is growing evidence that loss of caveolin-1 expression is associated with tumorigenesis [10-12]. Down-regulation or absence of caveolin-1 expression has been found in many human cancers, including primary breast, prostate, and colon cancers [13-15]. Furthermore, caveolin-1 null mice are more susceptible to carcinogen-induced tumorigenesis [10], suggesting that caveolin-1 may be a tumor suppressor.

There is accumulating experimental evidence *in vivo *and *in vitro *that caveolin-1 expression sensitizes cells to apoptotic stimulation. Elevated expression of endogenous caveolin-1 is associated with induction of apoptosis in mouse peritoneal macrophages [16]. Ectopic expression of caveolin-1 in NIH3T3 cells and T24 human bladder carcinoma cells sensitizes cells to staurosporine-induced apoptosis [17]. These data demonstrate that an up-regulation of caveolin-1 may be involved in promoting cell apoptosis.

In the present study, we investigated the effects of caveolin-1 on pituitary adenoma shrinkage in response to bromocriptine treatment at clinically-relevant concentrations in GH3 cells. Here we show that caveolin-1 in GH3 cells was up-regulated after bromocriptine treatment. Our data show that increased caveolin-1 expression sensitizes pituitary adenoma GH3 cells to apoptosis induced by bromocriptine treatment and clarifies the molecular mechanism of bromocriptine therapy of pituitary adenoma.

## Results

### Ectopic expression of recombinant caveolin-1 in GH3 cells results in apoptotic phenotypes

**Figure 1:**
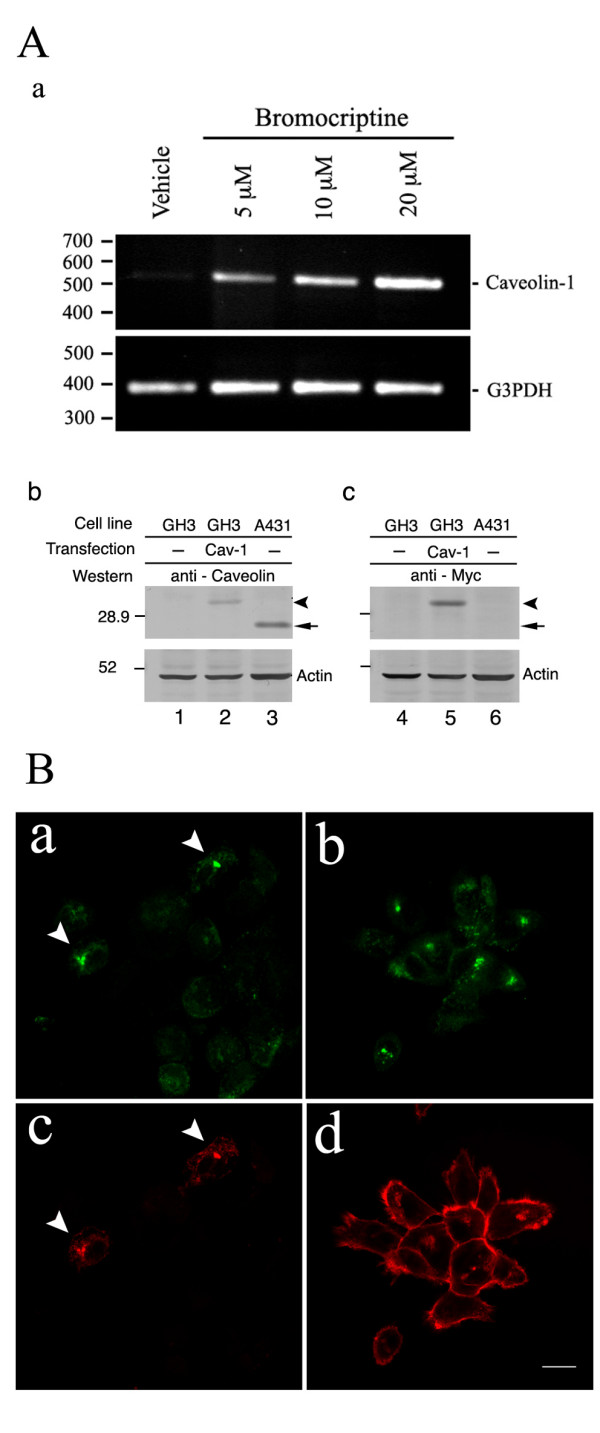
**Bromocriptine enhances endogenous caveolin-1 mRNA expression in GH3 cells.** (A) Panel a: Expression of caveolin-1 mRNA was enhanced after bromocriptine treatment. Panel b and c: Cellular proteins were extracted and Western blots performed 48 hours after recombinant caveolin-1 was transfected into GH3 cells (lane 2 and 5). Vehicle transfections (lane 1 and 4) were used as controls. Panel b is an immunoblot using rabbit anti-caveolin-1 antibody and panel c is an immunoblot using monoclonal anti-Myc antibody against Myc-tagged caveolin-1. Arrowhead indicates Myc-tagged caveolin-1. Arrow indicates endogenous caveolin-1. Protein extracted from A431 cells was used as an immunoblotting positive control. (B) Recombinant caveolin-1 expressed in A431 cells has identical perinuclear localization with endogenous caveolin-1. Myc-tagged caveolin-1 was transfected into A431 cells (a, c) for 48 hours. Non-transfected A431 cells were used to examine endogenous caveolin-1 localization (b, d). Cells were fixed and immunocytochemically stained with anti-caveolin-1 antibody then visualized by anti-rabbit IgG conjugated-FITC secondary antibody to detect exogenous (a) and endogenous (b) caveolin-1. Monoclonal anti-Myc antibody combined with anti-mouse IgG conjugated-Texas-Red was used to recognize recombinant caveolin-1 (c). Filamentary actin was stained with phalloidin conjugated-Texas-Red to observe cell morphology (d). Scale bar = 20 μm.

**Figure 2:**
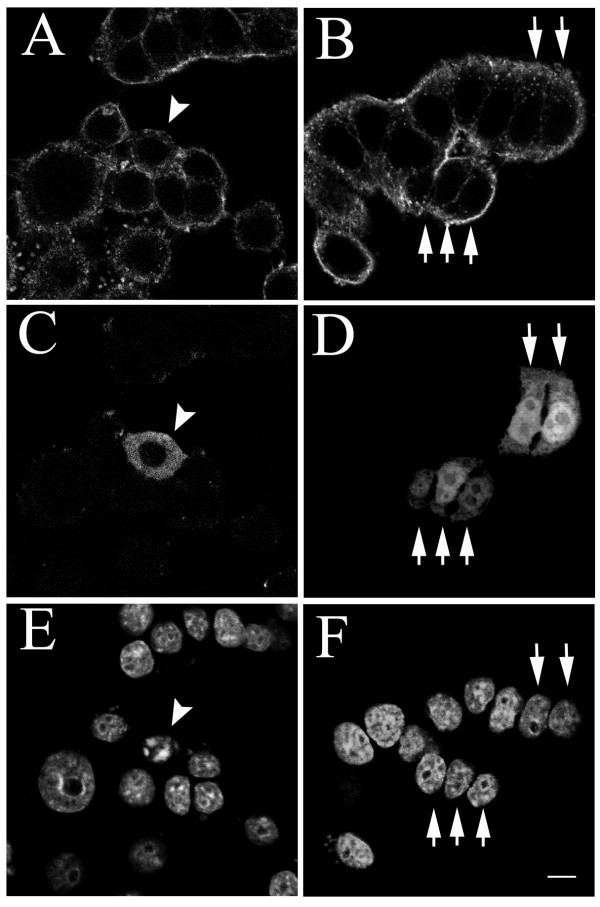
Overexpression of caveolin-1 induces nuclear condensation in GH3 cells. GH3 cells were transfected with either pcDNA4-caveolin-1 (A, C and E) or pcDNA4-EGFP (B, D and F). Immunocytochemical staining was carried out 48 hours after transfection. Phalloidin conjugated-Texas-Red (A and B) and Hoechst 33342 (E and F) dyes were used to stain F-actin and nuclei respectively. Cells expressing caveolin-1 (arrowhead) were visualized by anti-rabbit IgG conjugated-FITC secondary antibody (C). Caveolin-1 overexpressing cells exhibit nuclear condensation (E), while normal nuclei (arrow) were detected in cells over-expressing EGFP (F). Scale bar = 20 μm.

**Figure 3:**
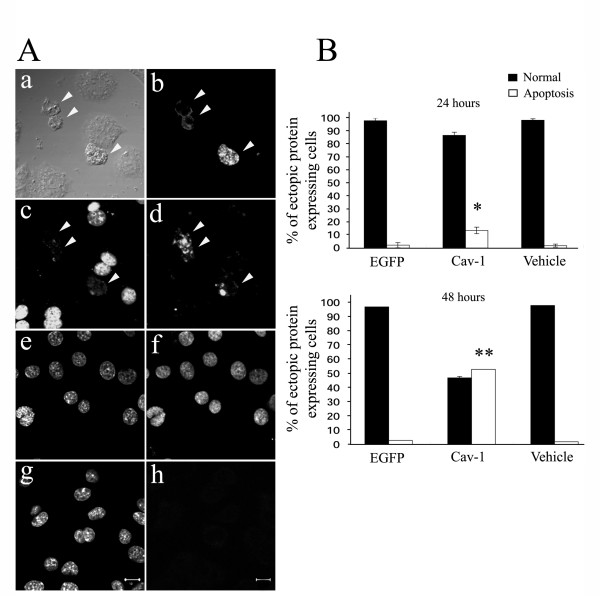
Over-expression of caveolin-1 induces apoptosis of GH3 cells. (A) GH3 cells were transfected with pcDNA4-Caveolin-1 for 48 hours then subjected to TUNEL assays. Cells transiently expressing exogenous caveolin-1 (indicated by arrowheads) showed shrunken morphologies (a and b) and nuclear fragmentation when stained with Hoechst 33342 dye (c) and were positively fluorescence-labeled by TUNEL assay (d). Cells directly treated with DNase I (e, f) or vehicle (g, h) were TUNEL positive (f) or negative (h), respectively. Nuclei stained with Hoechst 33342 dye are shown in c, e and g. (B) Caveolin-1 elicited apoptosis of GH3 cells. GH3 cells were transfected with pcDNA4-caveolin-1, pcDNA4-EGFP or vehicle (null treatment) and subjected to immunocytochemical staining 24 and 48 hours after transfection. Three hundred cells were randomly chosen and counted in each experiment (vehicle, caveolin-1 or EGFP) to determine the percentage with fragmented nuclei after Hoechst 33342 dye labeling. Apoptotic cells were measured by fluorescence labeling and data were expressed as mean ± standard deviation from n = 3 independent experiments (angular transformed for analysis, back-transformed for presentation). The standard deviations are too small to observe in the 48 hours data. **P < 0.01 and *P < 0.05 versus EGFP or vehicle experiment.

Caveolin-1 is associated with apoptosis and has been detected in GH3 cells [18]. As bromocriptine stimulates GH3 cell shrinkage and apoptosis, we hypothesized that bromocriptine treatment would induce GH3 cell apoptosis via caveolin-1. Semi-quantitative RT-PCR was used to detect the amount of caveolin-1 mRNA in rat GH3 cells before and after bromocriptine administration at different dosages according previous report [4]. Caveolin-1 mRNA was elevated after 24 hours of bromocriptine treatment in a dose-dependent manner (Fig. 1A,a). To explore the function of caveolin-1 in GH3 cells, a pcDNA4-Caveolin-1 plasmid containing Myc-tagged mouse caveolin-1 under the control of the CMV promoter was constructed and successfully transfected into GH3 cells. A predicted 30 kDa recombinant protein was recognized by an anti-caveolin-1 antibody (Fig. 1A,b) and by an anti-Myc antibody (Fig. 1A,c). Twenty-four kDa endogenous caveolin-1 expressed in the A431 cell line, and recognized by an anti-caveolin-1 antibody, was used as the positive control in Western blotting (Fig. 1A,b). We next clarified whether the recombinant caveolin-1 was localized in cells in the same manner as endogenous caveolin-1. The distribution of endogenous caveolin-1 in A431 cells was determined by immunofluorescent staining with an anti-caveolin-1 polyclonal antibody. Exogenous Myc-tagged caveolin-1 distribution in A431 cells was detected by double immunofluorescent staining with anti-caveolin-1 and anti-Myc polyclonal antibodies. Both endogenous and exogenous caveolin-1 had the same punctate distribution in the perinuclear region (Fig. 1B,a and 1B,b). We then transiently expressed the Myc-tagged caveolin-1 in GH3 cells to examine the effect of caveolin-1 on GH3 cells. By 48 hours after caveolin-1 expression in GH3 cells, apoptosis-like nuclear condensation was visible upon staining with Hoechst 33342 (bisbenzimide, Fig. 2E). In the control experiment, transient expression of enhanced-green fluorescent protein (EGFP) did not alter nuclear morphology (Fig. 2F). These data indicate that transient expression of Caveolin-1 induces apoptosis in GH3 cells. To test this, the TUNEL assay, which discriminates apoptosis from necrosis and from primary DNA strand breaks, was used to measure DNA fragmentation 48 hours after transfection [19]. Recombinant caveolin-1-expressing cells appeared shrunken with positive TUNEL labeling (Fig. 3A,d). In the control experiments, all cells were positively TUNEL labeled after exposure to DNase I (Fig. 3A,f), whereas no cell was TUNEL labeled after vehicle treatment (Fig. 3A,h). We quantified the proportion of apoptotic cells in ectopic caveolin-1 or EGFP-expressing cells by counting the number of cells exhibiting nuclear condensations per 100 exogenous caveolin-1- or EGFP-expressing cells, stained with Hoechst 33342. Significant numbers of caveolin-1 expressing cells exhibited apoptosis after transfection (13% at 24 and 53% at 48 hours), whereas only 2% and 3% of EGFP-expressing cells and 1.6–2% vehicle-treated cells exhibited apoptosis 24 and 48 hours after transfection (Fig. 3B). This result revealed that even transient caveolin-1 expression increased GH3 cell apoptosis.

### Caveolin-1 induced apoptosis of GH3 cells involves caspase 8

**Figure 4:**
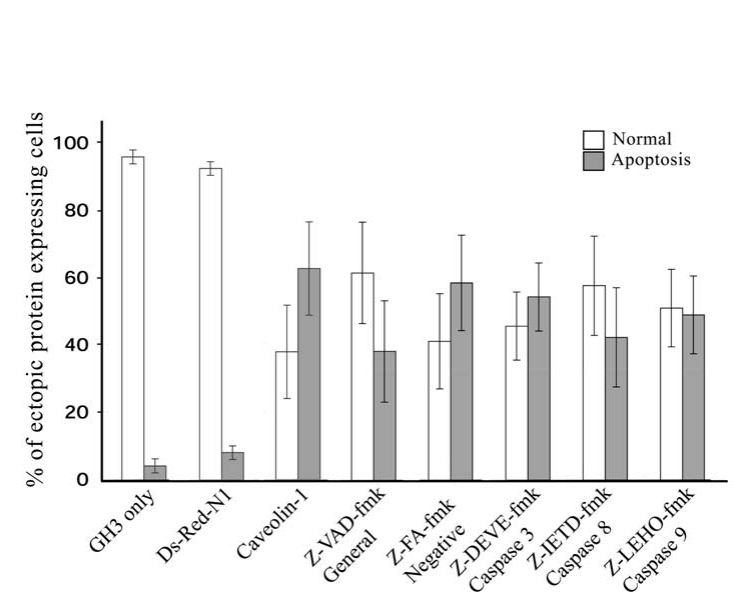
Caveolin-1 induces GH3 apoptosis via caspase 8 activity. Caspase inhibitors (50 μM) were added to either pcDNA4-Caveolin-1 or pcDNA4-DsRed-N1 (negative control) transfected GH3 cells and the proportion of apoptotic cells quantified 48 hours after transfection by immunocytochemical and TUNEL assays. Data were expressed as mean ± standard deviation from 3 independent experiments (angular transformed for analysis, back-transformed for presentation). Abbreviations on the x-axis: General caspase inhibitor (Z-VAD-fmk); a negative control inhibitor (Z-FA-fmk); specific caspase inhibitors for caspase 3 (Z-DEVE-fmk), caspase 8 (Z-IETD-fmk) or caspase 9 (Z-LEHO-fmk).

The activation of caspases plays a pivotal role in the execution of apoptosis by various signaling pathways [20,21]. To examine the role of caspases in apoptotic GH3 cells after transient caveolin-1 expression (Fig. 4), we separately treated the ectopic caveolin-1 and DsRed-N1 expressing GH3 cells with a general caspase inhibitor (Z-VAD-fmk), as well as specific caspase inhibitors, for caspase 3 (Z-DEVE-fmk), caspase 8 (Z-IETD-fmk) or caspase 9 (Z-LEHO-fmk) before determining the number of apoptotic cells by TUNEL assay. Over-expression of caveolin-1 resulted in 62% of the transiently-transfected cells becoming apoptotic. This effect was inhibited by treating GH3 cells with the general caspase inhibitor Z-VAD-fmk (24% apoptotic caveolin-1 expressing cells) and with the caspase 8 specific inhibitor, Z-IETD-fmk, (20% apoptosis). Treatment with caspase 3 or caspase 9 specific inhibitors did not inhibit caveolin-1 induced apoptosis (apoptosis decreased by 8 and 13%, respectively; *P *> 0.05). In negative control experiments, cells expressing the red fluorescent protein, DsRed-N1 had no significant increase in apoptosis compared to untreated GH3 cells (8% versus 2%, respectively). Treatment of GH3 cells with Z-FA-fmk, a negative control caspase inhibitor reagent, however, did not significantly inhibit caveolin-1 induced apoptosis (59% of cell apoptosis), indicating that activation of caspases plays a role in caveolin-1 induced GH3 cell apoptosis, at least in part through caspase 8 signaling.

### Bromocriptine sensitizes the caveolin-1 induced apoptosis in GH3 cells

**Figure 5:**
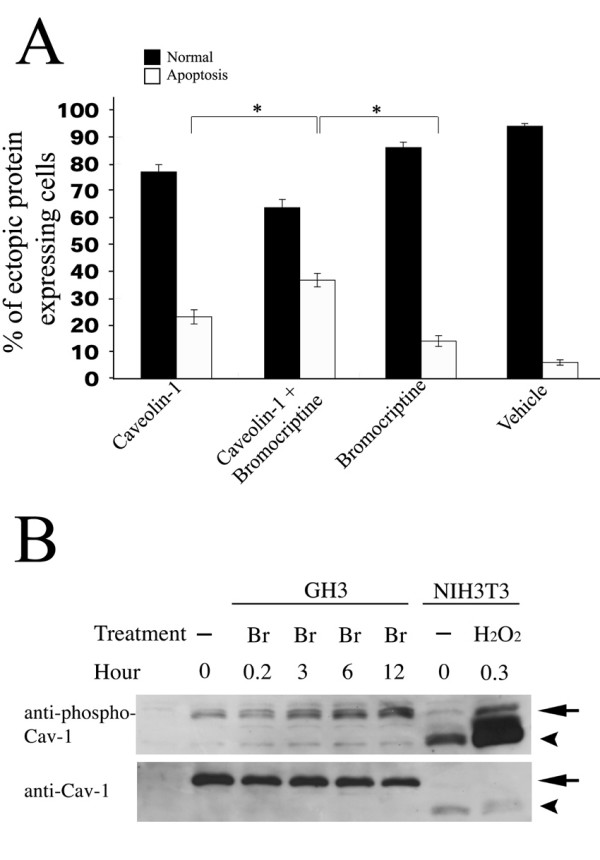
A combination of bromocriptine treatment and overexpression of Caveolin-1 enhances apoptosis of GH3 cells. (A) GH3 cells were transfected with caveolin-1 for 24 hours and then treated with or without 30 μM bromocriptine for another 12 hours. Non-transfected cells were treated for 12 hours with ethanol alone (vehicle) or bromocriptine. Cells expressing exogenous Caveolin-1 were immunostained with anti-Myc antibody and then detected with anti-mouse IgG Texas Red-conjugated secondary antibody. Three hundred Caveolin-1 expressing cells were counted in each experiment and apoptotic cells were determined by nuclear fragmentation after staining with Hoechst 33342 dye. Data were expressed as mean ± standard deviation from 3 independent experiments (angular transformed for analysis, back-transformed for presentation). (B) Phosphorylation of Tyr14 was enhanced when GH3 cells were exposed to bromocriptine. Cells were administered 30 μM bromocriptine as indicated, 24 hours after pcDNA4-caveolin-1 transfection. Cellular protein was extracted and separated on 12% SDS-PAGE then subjected to Western blotting. Anti-caveolin-1 or anti-phosphorylated caveolin-1 (Tyr^14^) specific antibodies were used to measure total and phosphorylated caveolin-1 respectively. Cellular protein extracts from NIH3T3 cells exposed to H_2_O_2 _were used as a positive control to detect phosphorylated caveolin-1. Dash (-) indicates vehicle treatment. The arrowhead points to endogenous caveolin-1 and the arrow marks recombinant caveolin-1. Abbreviation: Br, bromocriptine. *P < 0.05 versus experiment for caveolin-1 transient expression only or bromocriptine treatment only.

Bromocriptine induces activation of p38 MAP kinase in GH3 cells [12]. Activation of the p38 MAP kinase signaling pathway in NIH3T3 cells causes phosphorylation of caveolin-1 on Tyr^14 ^[22]. We examined if there was any relationship between bromocriptine and caveolin-1 that affected GH3 cell apoptosis. GH3 cells were allowed to transiently express caveolin-1 for 24 hours, then 30 μM bromocriptine was added for another 12 hours. The proportion of apoptotic cells was determined by estimating the number of cells containing condensed nuclear DNA. After 12 hours of bromocriptine treatment, the number of apoptotic cells was 38% of the total caveolin-1 expressing cell population compared to 24% without bromocriptine (Fig. 5A). Only 14% and 6% of the total cell population underwent apoptosis when cells were treated with bromocriptine or vehicle for 12 hours (Fig. 5A). The data indicate that there is an interaction between caveolin-1 and bromocriptine in the induction of GH3 apoptosis.

### Bromocriptine enhances phosphorylation of caveolin-1 Tyr^14^

Tyrosine^14 ^phosphorylation of caveolin-1 was found to be associated with apoptosis in the human promyelocytic leukemia cell line HL-60 after etoposide induction [23], indicating phosphorylation of caveolin-1 tyrosine may play a role in apoptosis. To explore whether bromocriptine could induce caveolin-1 phosphorylation, GH3 cells were transfected with pcDNA4-caveolin-1 for 24 hours, then exposed to bromocriptine as indicated in figure 5B. Total proteins were extracted, separated using SDS-PAGE and examined by Western blotting using an antibody specific for caveolin-1 phosphorylated at Tyr^14^. After 12 hours of bromocriptine treatment the amount of caveolin-1 phosphorylation was 3.75 times higher than with vehicle (ethanol) treatment (Fig. 5B). Cellular protein from H_2_O_2_-exposed NIH3T3 cells was used as a positive control for phosphorylated caveolin-1. These data demonstrated that bromocriptine enhanced phosphorylation of caveolin-1 in GH3 cells.

## Discussion

In the present study, we demonstrated GH3 cells overexpressing caveolin-1 underwent apoptosis. Caveolin-1 has previously been reported to be associated with enhancement of apoptotic sensitivity. For example, NIH3T3 cells treated with anti-sense caveolin-1 were resistant to staurosporine-induced apoptosis, and T24 bladder carcinoma cells were sensitized to caspase 3 activation when stably expressing recombinant caveolin-1 [17]. Large increases in caveolin-1 expression have also been observed in apoptotic-agent-treated mouse peritoneal macrophages [16]. In addition, retrovirus insertion-mediated random mutagenesis applied to L292 cells revealed caveolin-1 is necessary for apoptosis induced by TNF-α [24]. These reports highlight a growing body of evidence indicating that up-regulated expression of caveolin-1 is associated with cell apoptosis. This study has now shown, for the first time, that caveolin-1 itself induces apoptosis of rat anterior pituitary epithelial cells of the GH3 cell line.

We have shown that caveolin-1 mRNA expression was further enhanced in GH3 cells after 24 hours of bromocriptine stimulation. It is known that with bromocriptine stimulation of GH3 cells leads to accumulation of wild type p53 [25]. There are two consensus half sites (-292 to -283 bp and -273 and -264 bp) in the caveolin-1 promoter that are predicted to be wild type p53 binding sites. Previous experiments have also shown that transfection of wild type p53 into human skin fibroblasts is accompanied by a six-fold increase in caveolin-1 promoter transcription activity [26]. Therefore, and supported by evidence from this study, we speculate that bromocriptine enhances caveolin-1 expression via an increase in wild-type p53 expression in GH3 cells.

Caspases play critical roles in the initiation and execution of the apoptotic process [20]. We found that, at least in part, caspase 8 might be required for caveolin-1 induction of GH3 cell apoptosis. In contrast, up-regulation of caveolin-1 sensitizes NIH3T3 fibroblasts and T24 bladder carcinoma epithelial cells to apoptotic stimuli via increased activation of caspase 3 [17]. Caveolin-1 up-regulation is also associated with simvastatin-induced apoptosis of thioglycollate-elicited mouse peritoneal macrophages; this effect was independent of caspase activity, because the general caspase inhibitor Z-VAD-Fmk failed to block cell death [16]. Together these results indicate caveolin-1 mediates cellular apoptosis through variant signaling pathways in different cell types.

Treatment of lymphocytes and NIH3T3 cells with H_2_O_2 _or UV light induces phosphorylation of caveolin-1 Tyr^14 ^[27,17]. In addition, caveolin-1 phosphorylated at Tyr^14 ^specifically co-localizes with paxillin at focal adhesion complexes after epidermal growth factor, insulin or H_2_O_2 _treatment, suggesting phosphorylation of caveolin-1 is associated with cellular morphological changes and shrinkage when cells adapt to external cellular stresses [22,27,28]. In addition, etoposide, a therapeutic agent for leukemia, in inducing tumor cell apoptosis, increases phosphorylation of caveolin-1 Tyr^14 ^in HL-60 cells [23]. We demonstrated that phosphorylation of caveolin-1 Tyr^14 ^was increased after bromocriptine treatment and this accelerated GH3 cells apoptosis, indicating phosphorylation of caveolin-1 Tyr^14 ^may be important for bromocriptine mediation of cellular apoptosis or morphological changes in cells encountering apoptotic stress.

## Conclusion

In summary, the present results demonstrate that caveolin-1 expression is induced after bromocriptine treatment in rat pituitary adenoma cells. Moreover, exogenous overexpression of caveolin-1 increases apoptosis of pituitary adenoma cells and enhances bromocriptine-induced cell apoptosis. Interestingly, caveolin-1 was phosphorylated at Tyr^14 ^when GH3 cells responded to bromocriptine treatment. Phosphorylation of caveolin-1 Tyr^14 ^is predicted to be associated with cellular apoptosis. These data suggest that bromocriptine-induced pituitary adenoma cell apoptosis may result from enhanced expression and activation of caveolin-1 via increased caveolin-1 phosphorylation. Our result explains the therapeutic effect of bromocriptine in curing pituitary adenoma.

## Materials and methods

### Materials and reagents

Dulbecco's modified Eagle medium, penicillin, streptomycin, L-glutamine, fetal bovine serum and horse serum were purchased from Life Technologies (Gaithersburg, MD). Rabbit anti-caveolin-1 and rabbit anti-phosphorylated caveolin-1 (Tyr^14^) antibody were bought from Chemicon Internal Inc. (Temecula, CA). Mouse anti-c-Myc-antibody (9E10) was obtained from Santa Cruz Biotechnology Inc. (Santa Cruz, CA). Texas-Red and FITC conjugated secondary antibodies and normal goat serum were purchased from Jackson ImmunoResearch Laboratories (West Grove, PA).

### Plasmid construction and semi-quantitated RT-PCR

Total RNA was extracted from adult C57Bl/6 mouse brain with TRIzol reagent (Invitrogen, Carlsbad, CA) according to the manufacturer's instructions. Caveolin-1 cDNA was amplified from total RNA by RT-PCR and was subcloned into pGEM T-easy vector by TA cloning (Promega, Madison, WI). The paired primer sequences used for RT-PCR were as follows: forward primer, 5'-CTCGAGATGTCTGGGGGCAAATACGTG-3'; reverse primer, 5'-TCTAGATATCTCTTTCTGCGTGCTGATGCG-3'. The DNA sequence of caveolin-1 was confirmed by auto-sequencing using an ABI 3730 autosequenser. Caveolin-1 in the pGEM T-easy vector was digested with *Xho *I and *Xba *I restriction enzymes and then subcloned into pcDNA4 (Invitrogen, Carlsbad, CA) mammalian expression vector; this plasmid was termed pcDNA4-caveolin-1. pcDNA4-EGFP was cloned as follows: the clone pEGFP-N1 plasmid containing enhanced green fluorescent protein (EGFP), obtained from Clontech (Palo Alto, CA), was digested with *Pst *I and *Not *I restriction enzymes, then subcloned into pcDNA4 vector; this plasmid was designated pcDNA4-EGFP. pDsRed-N1 containing red fluorescent protein was purchased from Clontech Laboratories. For quantifying the level of caveolin-1 cDNA expressed in GH3 cells modulated after bromocriptine treatment, the cells were treated with either bromocriptine (Sigma) at different concentrations of 5, 10, 20 μM or with vehicle for 24 hours, when total RNA was extracted and the transcripts quantified by RT-PCR, as described above.

### Cell culture

The rat GH3 pituitary adenoma and human A431 epithelial cell lines were obtained from the American Type Cell Collection. The GH3 cells were propagated in F12K nutrient mix medium (Invitrogen, Carlsbad, CA) supplemented with 2.5% fetal bovine serum, 15% horse serum, 2 mM L-glutamine, 100 units/ml penicillin, and 100 units/ml streptomycin. The A431 cells were maintained in DMEM medium containing 10% fetal bovine serum, 2 mM L-glutamine, 100 units/ml penicillin, and 100 units/ml streptomycin.

### Transfections

Transfection of GH3 and A431 cells was performed using Lipofectamine Plus™ reagent (Invitrogen, Carlsbad, CA) according to the manufacturer's protocol. Briefly, the day before transfection, 6 × 10^5 ^cells were plated on a 6-well cell culture grade Petri dish. One μg DNA and 6 μl Plus™ reagent were diluted into 100 μl serum-free medium and 4 μl lipofectamine was added to 100 μl serum-free medium; these two pre-complexes were then mixed and incubated for 15 min at room temperature. The DNA-Plus™-lipofectamine reagent complex was added to each well containing GH3 or A431 cells in fresh serum-free medium. Cells were incubated at 37°C in 5% CO_2 _in air for 3 hours, then the old medium was replaced with fresh complete medium after incubation. The times after transfection for immunocytochemical staining or TUNEL (terminal deoxynucleotidyl transferase-mediated dUTP nick end-labeling) analyses are indicated in the results.

### Immunocytochemistry

For the analysis of Myc-tagged caveolin-1 expression in GH3 cells, cells were briefly washed with PBS and fixed with 4% paraformaldehyde in PBS for 15 min at room temperature. Cells were permeabilized by incubating with PBS containing 0.5% Triton X-100 for 10 min. The permeabilized cells were immersed in blocking solution containing 10% normal goat serum in PBS for 1 hour. The cells were then incubated over night at 4°C with either anti-caveolin-1 (diluted 1:100 in PBS) or Myc (diluted 1:100 in PBS) primary antibody. After three washes with PBS, cells were incubated with the secondary antibody (anti-mouse IgG conjugated with FITC, diluted 1:300 in PBS; anti-rabbit IgG conjugated with Texas-Red or FITC, diluted 1:300 in PBS) for 2 hours at room temperature. Slides were mounted with Mowiol 4–88 (Calbiochem, La Jolla, CA) and visualized by confocal laser scanning microscopy (Ziess, LSM510) before being digitally photographed.

### TUNEL assay

The TUNEL assays were conducted as previously described [2] with some modifications. Briefly, DNase I-treated GH3 cells or cells ectopically expressing caveolin-1 were washed twice with PBS and fixed with 4% paraformaldehyde in PBS, pH7.4, for 10 min at room temperature. Cells were permeabilized with 0.1% Triton X-100 in 0.1% sodium citrate for 2 min on ice. Cell ectopically expressing caveolin-1 were labeled with monoclonal anti-Myc antibody, then visualized by Texas-Red conjugated anti-mouse IgG antibody. Cells were TUNEL labeled using the *In situ *Cell Death Detection Kit (Roche, Alcaloid, Mannheim, Germany) according to the manufacturer's instruction.

### Caspase inhibitor treatment and quantification of cell apoptosis

Treatment with caspase inhibitors and quantification of cell apoptosis were conducted as follows: GH3 cells were seeded in a 24 well dish one day before transfection. Cells were transfected with pcDNA4-caveolin-1 or pDsRed-N1 by Lipofectamine Plus™ reagent. Transfected cells were treated with caspase inhibitors at 50 mM final concentration for 48 hours, then immunocytochemical and TUNEL assays were used to quantify apoptotic cells. Anti-c-Myc monoclonal antibody was used as the first antibody to recognize caveolin-1 expressing cells, followed by Texas Red-conjugated anti-mouse IgG. Cells expressing DsRed-N1 were directly detected by fluorescent microscopy. The caspase inhibitors Z-VAD-fmk, Z-DEVE-fmk, Z-IETD-fmk, and Z-LEHO-fmk, and the negative control Z-FA-fmk were purchased from Calbiochem Inc. One hundred Myc-tagged and DsRed-N1 expressing cells were examined in each experiment for positive TUNEL labeling. Each experiment was repeated three times. Data were analyzed using the GLM procedure of SAS (v9.1) and significant differences between means were determined by a Duncan's New Multiple Range Test (*P *< .05).

### Western immunoblotting

For detecting caveolin-1 and phosphorylated caveolin-1 protein expression in GH3 cells, cellular proteins were extracted with RIPA buffer (50 mM Tris-HCl, pH 7.0, 1% NP40, 0.25% sodium deoxycholate, 150 mM NaCl, 1 mM PMSF, 1 μg/ml aprotinin, 1 μg/ml leupeptine, 1 μg/ml peptasin A, 1 mM Na_3_VO_4_, 1 mM NaF, 10 mM Na_4_P_2_O_7_) 36 hours after pcDNA4-caveolin-1 transfection. Cell extracts were then centrifuged at 12000 rpm at 4°C for 10 min, and the supernatants collected. Protein samples were quantified by the Bio-Rad protein assay kit (cat. no. #500-0006; Bio-Rad, Inc., Hercules, CA). Each 10 μg sample was denatured for 5 min at 95°C in Laemmli sample buffer (2% SDS, 10% glycerol, 100 mM DTT, 60 mM Tris HCl, pH 6.8, 0.01% bromophenol blue). The proteins were then separated by 12% SDS-PAGE and transferred to PVDF membranes. Blots were washed with TBST buffer (120 mM Tris-HCl, pH 7.5, 150 mM NaCl, 0.05% Tween 20) then placed in TBST buffer supplemented with 5% skimmed milk powder for blocking non-specific interactions for 1 hour at room temperature. Blots were then incubated with rabbit anti-caveolin-1 antibody (1:1000 in TBST), mouse anti-Myc antibody (1:3000 in TBST) or anti-phosphorylated caveolin-1 (Tyr^14^) polyclonal antibody (1:1000 in TBST) overnight at 4°C. After washing the blots with TBST, membranes were incubated with horse-radish peroxidase-conjugated secondary antibodies (1:3000, Amersham Biosciences, Arlington Heights, IL) for 2 hours and washed again as described previously. Membrane-bound secondary antibodies were detected using the ECL procedure developed by Amersham Biosciences. To ensure equal protein loading membranes were rehybridized with a mouse anti-actin antibody (1:3000, Chemicon, Inc., Temecula, CA) and developed using X-ray film (Kodak).

### Statistical analysis

Bar charts of the proportion of apoptotic cells of each treatment group were drawn with the sample mean plus and minus one standard deviation from three independent experiments. The angular transformation of observed proportion data were used for statistical analysis. Analysis of variance of the group factor in blocks (treating each experiment as a block) and Tukey's Studentized range test for group means were performed with SAS v9.1 statistical analysis package (SAS Institute Inc., Cary, North Carolina).

## Authors' contributions

YNJ and YTJ conceived the study, participated in its design and helped to draft the manuscript. YHL carried out most of experiments. YNJ and YHL contributed equally toward this work. MWK and TYT performed TUNEL and apoptosis analysis. MCH, YBT, WTKC and YTJ participated in its design and helped to draft the manuscript. All authors read and approved the final manuscript.

## References

[B1] Molitch ME (2006). Prolactin-secreting tumors: what's new?. Expert Rev Anticancer Ther.

[B2] Colao A, Di Sarno A, Guerra E, De Leo M, Mentone A, Lombardi G (2006). Drug insight: Cabergoline and bromocriptine in the treatment of hyperprolactinemia in men and women. Nat Clin Pract Endocrinol Metab.

[B3] van de Weerdt C, Peers B, Belayew A, Martial JA, Muller M (2000). Far upstream sequences regulate the human prolactin promoter transcription. Neuroendocrinol.

[B4] Kanasaki H, Fukunaga K, Takahashi K, Miyazaki K, Miyamoto E (2000). Involvement of p38 mitogen-activated protein kinase activation in bromocriptine-induced apoptosis in rat pituitary GH3 cells. Biol Reprod.

[B5] Krajewska WM, Maslowska I (2004). Caveolins: structure and function in signal transduction. Cell Mol Biol Lett.

[B6] Engelman JA, Chu C, Lin A, Jo H, Ikezu T, Okamoto T, Kohtz DS, Lisanti MP (1998). Caveolin-mediated regulation of signaling along the p42/44 MAP kinase cascade in vivo. FEBS lett.

[B7] Shridhar V, Sun QC, Miller OJ, Kalemkerian GP, Petros J, Smith DI (1997). Loss of heterozygosity on the long arm of human chromosome 7 in sporadic renal cell carcinomas. Oncogene.

[B8] Singh RD, Puri V, Valiyaveettil JT, Marks DL, Bittman R, Pagano RE (2003). Selective Caveolin-1-dependent endocytosis of glycosphingolipids. Mol Biol Cell.

[B9] van Deurs B, Roepstorff K, Hommelgaard AM, Sandvig K (2003). Caveolae: anchored, multifunctional platforms in the lipid ocean. Trends Cell Biol.

[B10] Capozza F, Williams TM, Schubert W, McClain S, Bouzahzah B, Sotgia F, Lisanti MP (2003). Absence of Caveolin-1 sensitizes mouse skin to carcinogen-induced epidermal hyperplasia and tumor formation. Am J Pathol.

[B11] Koleske A, Baltimore D, Lisanti MP (1995). Reduction of caveolin and caveolae in oncogenically transformed cells. Proc Natl Acad Sci USA.

[B12] Lee SW, Reimer CL, Oh P, Campbell DB, Schnitzer JE (1998). Tumor cell growth inhibition by caveolin re-expression in human breast cancer cells. Oncogene.

[B13] Jenkins R, Takahashi S, DeLacey K, Bergstralh E, Lieber M (1998). Prognostic significance of allelic imbalance of chromosome arms 7q, 8p, 16q, and 18q in stage T_3_N_0_M_0 _prostate cancer. Genes Chromosomes Cancer.

[B14] Zenklusen JC, Bieche I, Lidereau R, Conti CJ (1994). (C-A)n microsatellite repeat D7S522 is the most commonly deleted region in human primary breast cancer. Proc Natl Acad Sci USA.

[B15] Zenklusen JC, Thompson JC, Klein-Szanto AJ, Conti CJ (1995). Frequent loss of heterozygosity in human primary squamous cell and colon carcinomas at 7q31.1: evidence for a broad range tumor suppressor gene. Cancer Res.

[B16] Gargalovic P, Dory L (2003). Cellular apoptosis is associated with increased Caveolin-1 expression in macrophages. J Lipid Res.

[B17] Liu J, Lee P, Galbiati F, Kitsis RN, Lisanti MP (2001). Caveolin-1 expression sensitizes fibroblastic and epithelial cells to apoptotic stimulation. Am J Physiol Cell Physiol.

[B18] Freyberg Z, Bourgoin S, Shields D (2002). Phospholipase D2 is localized to the rims of the Golgi apparatus in mammalian cells. Mol Biol Cell.

[B19] Sgonc R, Gruber J Apoptosis detection: an overview. Exp Gerontol.

[B20] Earnshaw WC, Martins LM, Kaufmann SH (1999). Mammalian caspases: structure, activation, substrates, and functions during apoptosis. Annu Rev Biochem.

[B21] Philchenkov A (2004). Caspases: potential targets for regulating cell death. J Cell Mol Med.

[B22] Volonté D, Galbiati F, Pestell RG, Lisanti MP (2001). Cellular stress induces the tyrosine phosphorylation of Caveolin-1 (Tyr^14^) via activation of p38 mitogen-activated protein kinase and c-Src kinase. Evidence for caveolae, the actin cytoskeleton, and focal adhesions as mechanical sensors of osmotic stress. J Biol Chem.

[B23] Navakauskiene R, Treigyte G, Gineitis A, Magnusson KE (2004). Identification of apoptotic tyrosine-phosphorylated proteins after etoposide or retinoic acid treatment. Proteomics.

[B24] Ono K, Iwanaga Y, Hirayama M, Kawamura T, Sowa N, Hasegawa K (2004). Contribution of caveolin-1α and Akt to TNF-α-induced cell death. Am J Physiol Lung Cell Mol Physiol.

[B25] Yin D, Tamaki N, Kokunai T, Yasuo K, Yonezawa K (1999). Bromocriptine-induced apoptosis in pituitary adenoma cells: relationship to p53 and bcl-2 expression. J Clin Neurosci.

[B26] Bist A, Fielding CJ, Fielding PE (2000). p53 regulates caveolin gene transcription, cell cholesterol, and growth by a novel mechanism. Biochemistry.

[B27] Cao H, Sanguinetti AR, Mastick CC (2004). Oxidative stress activates both Src-kinases and their negative regulator Csk and induces phosphorylation of two targeting proteins for Csk: caveolin-1 and paxillin. Exp Cell Res.

[B28] Krump E, Nikitas K, Grinstein S (1997). Induction of tyrosine phosphorylation and Na^+^/H^+ ^exchanger activation during shrinkage of human neutrophils. J Biol Chem.

